# Response to Letter to the Editor: Concerns on modeling postmenopausal osteoporosis in young female rats

**DOI:** 10.1186/s13018-019-1485-2

**Published:** 2019-12-18

**Authors:** Juan Marcelo Rosales Rocabado, Masaru Kaku, Kosuke Nozaki, Takako Ida, Megumi Kitami, Yujin Aoyagi, Katsumi Uoshima

**Affiliations:** 1 Division of Bio-Prosthodontics, Niigata University Graduate School of Medical and Dental Sciences, Niigata, Japan; 2 Department of Biofunction Research, Institute of Biomaterials and Bioengineering, Tokyo Medical and Dental University, Tokyo, Japan

Dear Editor,

We appreciate Dr. Lelovas and Dr. Dontas’ interest and concerns regarding our manuscript, titled “A multi-factorial analysis of bone morphology and fracture strength of rat femur in response to ovariectomy,” which was recently published in the *Journal of Orthopaedic Surgery and Research* [1], and very much appreciate the opportunity to respond. In the study, we aimed to comprehensively evaluate changes in the bone architecture that influence its fracture strength in response to estrogen deficiency of the ovariectomized (OVX) rat model. The changes in bone architecture induced by OVX varied at different sites, especially between trabecular and cortical bones. The OVX increased the external diameter of the cortical bone with a concomitant increase in bone strength at the mid-diaphysis, while the trabecular bone volume was decreased at the distal-metaphysis. One of the concerns raised was that the increased breaking force at the mid-diaphysis after OVX might be a consequence of the use of young rats, as we used rats that were 10 weeks old at the time of OVX and analyzed them at 18 weeks old. Considering the remodeling status and microarchitecture of the rat cortical bone, the use of aged rats (> 9 months old) and long observation periods (> 6 months) have been recommended for osteoporosis studies [2, 3]. However, it was reported that OVX still increases the external diameter of long bones with a concomitant increase in bone strength even in 9-month-old rats [4]. Besides, due to the slow remodeling rate of the tissue, especially the cortical bone, sensitive detection approaches must be employed with aged-OVX models [5]. Note that fracture risk has not been reproduced in animal models, and the true degree of correspondence between bone changes in animal models and humans remains elusive [2]. Consequently, aged rats are not always suitable for the study of osteoporosis. While animal studies provide insight into understanding biological responses, special attention is needed when discussing the clinical relevance of the results obtained in animal studies.

Another concern involves the usage of a fixative to preserve specimens for the mechanical test. To precisely calculate the mechanical parameters of the bone, measurement of the bone architecture of each specimen prior to the mechanical test is crucial. In our experiment, we used microcomputed tomography images to analyze the dimensional parameters along with the bone mineral density (BMD). Scanning requires placing the samples at room temperature for a relatively long period of time. Thawing of frozen specimens, may cause protein degradation and thus, alterations in the mechanical properties of the tissue. Therefore, we chose to perform formaldehyde fixation for 3 days at 4 °C. Possible side effects of formaldehyde fixation are the formation of additional intra- and intermolecular crosslinks and the elution of inorganic components, as previously described [6]. However, a study reported that 2 weeks of formaldehyde fixation did not alter the elastic mechanical properties of murine bones [7]. It has also been reported that the BMD, as well as the initial Young’s modulus, show no significant differences between formaldehyde-fixed samples and frozen samples for up to 6 months of preservation [8]. Concerns about the use of fixed specimens for the mechanical test are mainly due to the long fixation periods; hence, the use of shorter fixation times can minimize side effects [7].

As pointed out, the experimental settings, including the age of the animals and the test conditions, can dramatically affect the study outcome. Although the use of aged rats may provide a model more closely resembling postmenopausal osteoporosis, there are drawbacks to this model as well. Thus, various approaches and comprehensive analyses are required to understand the etiology of multi-factorial bone disorders. Future studies using aged animals and longer observation periods will enhance our understanding of OVX-induced bone architectural changes, which is important to establish accurate bone fracture risk assessment in the clinic.

## Data Availability

Not applicable
